# Supplementary material for: A single-vesicle fluorescence microscopy platform to quantify phospholipid scrambling
Source: Nat Struct Mol Biol. 2026 Jun 15;33(6):1011–9. doi: 10.1038/s41594-026-01821-8 (PMC13275289; doi:10.1038/s41594-026-01821-8)
Supplement: Supplementary file 1 — Kinetic model for fitting single scramblase traces. [file 41594_2026_1821_MOESM1_ESM.pdf]

nature structural & molecular biology

Supplementary information https://doi.org/10.1038/s41594-026-01821-8

A single-vesicle fluorescence microscopy platform to quantify phospholipid scrambling

In the format provided by the authors and unedited

Appendix: Kinetic_model

A single vesicle fluorescence microscopy platform to quantify phospholipid scrambling

Sarina Veit[1], Grace I. Dearden[2], Kartikeya M. Menon[3], Faria Noor[2], Indu Menon[2], Takefumi Morizumi[4], Oliver P. Ernst[4,5,6], Anant K. Menon[2], Thomas Günther Pomorski[1,7]

[1]Department of Molecular Biochemistry, Faculty of Chemistry and Biochemistry, Ruhr University Bochum, Bochum 44801, Germany

[2]Department of Biochemistry and Biophysics, Weill Cornell Medicine, New York, New York, 10065, USA

[3]Icahn School of Medicine at Mount Sinai, New York, NY 10029

[4]Department of Biochemistry, [5]Department of Molecular Genetics, and [6]Department of Materials Science and Engineering, University of Toronto, Toronto, Ontario, Canada

[7]Department of Plant and Environmental Sciences, University of Copenhagen, DK-1871 Frederiksberg C, Denmark

Nomenclature

$L_{in}(t)$	Inner leaflet NBD lipid pool
$L_{out}(t)$	Outer leaflet NBD lipid pool
$L_{ex}(t)$	Extracted NBD lipid pool
α, β	Forward and backward scrambling rate constants
γ	Rate constant of NBD fluorophore extraction
$\mathbf{R}$	Coefficient matrix of $L_{in}(t)$ and $L_{out}(t)$ system
λ_i	Eigenvalues of $\mathbf{R}$
$\mathbf{v}_i$	Eigenvectors of $\mathbf{R}$
$\mathbf{I}_2$	Identity matrix dimension 2

1. Problem Definition

A three-compartment (L_{in}, L_{out}, L_{ex}) model is used to determine the rate constants for scrambling (α, β) and for buffer extraction (γ). The kinetic model for bilayer lipid distribution (inner leaflet: L_{in}, outer leaflet: L_{out}) and reduction (L_{ex}) is as follows:

$$\mathrm{L_{in}(t)} \underset{\beta}{\overset{\alpha}{\rightleftharpoons}} \mathrm{L_{out}(t)} \xrightarrow{\gamma} \mathrm{L_{ex}} \tag{1}$$

The evolution in time t of the three-compartment model is defined with a system of differential equations:

$$\frac{\mathrm{d}L_{in}(t)}{\mathrm{d}t} = L'_{in}(t) = -\alpha L_{in}(t) + \beta L_{out}(t) \tag{2}$$

$$\frac{\mathrm{d}L_{out}(t)}{\mathrm{d}t} = L'_{out}(t) = \alpha L_{in}(t) + (-\beta - \gamma) L_{out}(t) \tag{3}$$

$$\frac{\mathrm{d}L_{ex}(t)}{\mathrm{d}t} = L'_{ex}(t) = \gamma L_{out}(t) \tag{4}$$

The following initial conditions are assumed:

$$L_{ex}(t=0) = 0; L_{in}(t=0) + L_{out}(t=0) = 1 \tag{5}$$

To solve this 3-compartment model, only equations (2) and (3) need to be considered, as the formation of $L_{ex}(t)$ is independent of $L_{in}(t)$ and does not affect the dynamics of the 2×2 system. (If preferred, in the equivalent formulation in terms of a Markov chain, $L_{ex}(t)$ represents an absorbing state and doesn't need to be solved directly).

This system can be represented in matrix form:

$$\begin{pmatrix} L'_{in}(t) \\ L'_{out}(t) \end{pmatrix} = \begin{pmatrix} -\alpha & \beta \\ \alpha & -(\beta+\gamma) \end{pmatrix} \begin{pmatrix} L_{in}(t) \\ L_{out}(t) \end{pmatrix} \tag{6}$$

This is a 2×2 homogeneous system of first order ordinary differential equations, alternatively shown as: $\mathbf{L}'(t) = \mathbf{R}\mathbf{L}(t)$. The general solution takes the form:

$$\mathbf{L}(t) = \begin{pmatrix} c_1 v_{11} e^{\lambda_1 t} + c_2 v_{12} e^{\lambda_2 t} \\ d_1 v_{21} e^{\lambda_1 t} + d_2 v_{22} e^{\lambda_2 t} \end{pmatrix} = \begin{pmatrix} A_1 e^{\lambda_1 t} + A_2 e^{\lambda_2 t} \\ B_1 e^{\lambda_1 t} + B_2 e^{\lambda_2 t} \end{pmatrix} \tag{7}$$

2. Computing Eigenvalues and Eigenvectors

Eigenvalues of the homogeneous system are the roots of the characteristic equation, solved below:

$$\det(\mathbf{R} - \lambda \mathbf{I}_2) = \begin{vmatrix} -\alpha - \lambda & \beta \\ \alpha & -(\beta + \gamma + \lambda) \end{vmatrix} = 0 \tag{8}$$

$$(-\alpha - \lambda)(-\beta - \gamma - \lambda) - \alpha\beta = 0 \tag{9}$$

$$\lambda^2 + (\alpha + \beta + \gamma)\lambda + \alpha\gamma = 0 \tag{10}$$

$$\lambda = \frac{-(\alpha + \beta + \gamma) \pm \sqrt{(\alpha + \beta + \gamma)^2 - 4\alpha\gamma}}{2} \tag{11}$$

Eigenvectors $\mathbf{v}_i$ arise from the 2×2 system

$$(\mathbf{R} - \lambda_i \mathbf{I}_2)\mathbf{v}_i = \mathbf{0}$$

Where v_{11} and v_{21} are components of eigenvector $\mathbf{v}_1$, the example equation below can be solved for both eigenvectors.

$$(-\alpha - \lambda_1)v_{11} + \beta v_{21} = 0$$
$$\alpha v_{11} + (-\beta - \gamma - \lambda_1)v_{21} = 0$$

$$(\mathbf{v}_1 \mathbf{v}_2) = \begin{pmatrix} \beta & \beta \\ \alpha + \lambda_1 & \alpha + \lambda_2 \end{pmatrix} \tag{12}$$

These eigenvectors can be equivalently computed by substituting equation (7) into the original system of differential equations (6):

$$\begin{pmatrix} L'_{in}(t) \\ L'_{out}(t) \end{pmatrix} = \begin{pmatrix} \lambda_1 A_1 e^{\lambda_1 t} + \lambda_2 A_2 e^{\lambda_2 t} \\ \lambda_1 B_1 e^{\lambda_1 t} + \lambda_2 B_2 e^{\lambda_2 t} \end{pmatrix} = \begin{pmatrix} -\alpha & \beta \\ \alpha & -(\beta + \gamma) \end{pmatrix} \begin{pmatrix} A_1 e^{\lambda_1 t} + A_2 e^{\lambda_2 t} \\ B_1 e^{\lambda_1 t} + B_2 e^{\lambda_2 t} \end{pmatrix} \tag{13}$$

Simplifying this equation leads to equivalent eigenvector construction as in the solution above:

$$A_1(\lambda_1 + \alpha)e^{\lambda_1 t} + A_2(\lambda_2 + \alpha)e^{\lambda_2 t} = \beta B_1 e^{\lambda_1 t} + \beta B_2 e^{\lambda_2 t} \tag{14}$$

$$B_1 = A_1 \frac{\lambda_1 + \alpha}{\beta} \tag{15}$$

$$B_2 = A_2 \frac{\lambda_2 + \alpha}{\beta} \tag{16}$$

For more compact notation, f_i is defined:

$$f_i = \frac{\alpha + \lambda_i}{\beta} \tag{17}$$

3. Solving Initial Conditions

The remaining unknowns in the expression of bilayer lipid concentrations are A_1 and A_2. These variables are calculated using the initial condition assumptions laid out in Section

1.

$$L_{in}(t) = A_1 e^{\lambda_1 t} + A_2 e^{\lambda_2 t} \tag{18}$$

$$L_{out}(t) = B_1 e^{\lambda_1 t} + B_2 e^{\lambda_2 t} = \frac{\alpha + \lambda_1}{\beta} A_1 e^{\lambda_1 t} + \frac{\alpha + \lambda_2}{\beta} A_2 e^{\lambda_2 t} \tag{19}$$

$$L_{in}(t=0) + L_{out}(t=0) = 1 \tag{20}$$

A_1 and A_2 can be solved by substitution:

$$L_{in}(t=0) = A_1 + A_2 \tag{21}$$

$$L_{out}(t=0) = B_1 + B_2 = f_1 A_1 + f_2 A_2 \tag{22}$$

$$L_{out}(t=0) = f_1(L_{in}(t=0) - A_2) + f_2 A_2 \tag{23}$$

$$L_{out}(t=0) - f_1 L_{in}(t=0) = A_2(f_2 - f_1) \tag{24}$$

$$\tag{25}$$

$$A_2 = \frac{f_1 L_{in}(t=0) - L_{out}(t=0)}{f_1 - f_2} \tag{26}$$

$$A_1 = \frac{L_{out}(t=0) - f_2 L_{in}(t=0)}{f_1 - f_2} \tag{27}$$

As the reaction between L_{out} and L_{ex} is irreversible, computing an additional term for A_1 and A_2 for such an inhomogeneous system is not necessary.

4. Modeling

The final formulae used for regression incorporate all these derived terms and the initial condition assumption in (5) that fluorophores are split between the inner and outer leaflets at time $t = 0$.

$$L_{in}(t) = \frac{1 - f_2}{2(f_1 - f_2)} e^{\lambda_1 t} + \frac{f_1 - 1}{2(f_1 - f_2)} e^{\lambda_2 t} \tag{28}$$

$$L_{out}(t) = \frac{f_1 - f_1 f_2}{2(f_1 - f_2)} e^{\lambda_1 t} + \frac{f_1 f_2 - f_2}{2(f_1 - f_2)} e^{\lambda_2 t} \tag{29}$$

Using Python and SciPy, the total fluorescence $F(t) = L_{in}(t) + L_{out}(t)$ can be fitted against time t with nonlinear least squares to identify α, β, and γ (in our model we set $\alpha = \beta$). As each experiment has 10 data points, $n/10$ parameters can be reasonably fit for bias-variance trade-off, so a univariate fit for α was performed. The initial split of fluorophores between the inner and outer leaflets was allowed to vary between 0.40 and 0.60 in the univariate model for α.
