# Supplementary figures and images for: A single-vesicle fluorescence microscopy platform to quantify phospholipid scrambling
Source: Nat Struct Mol Biol. 2026 Jun 15;33(6):1011–9. doi: 10.1038/s41594-026-01821-8 (PMC13275289; doi:10.1038/s41594-026-01821-8)

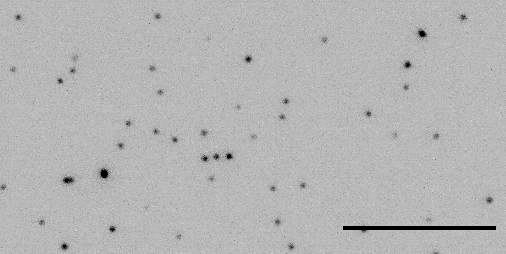

Supplement: Supplementary file 4 — Original microscopy image for Fig. 1c. [file 41594_2026_1821_MOESM4_ESM.tif]

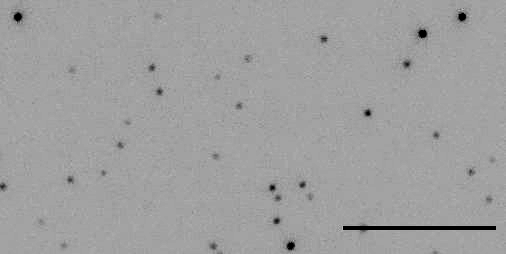

Supplement: Supplementary file 5 — Original microscopy image for Fig. 1c. [file 41594_2026_1821_MOESM5_ESM.tif]
