# Supplementary material for: A single-vesicle fluorescence microscopy platform to quantify phospholipid scrambling
Source: Nat Struct Mol Biol. 2026 Jun 15;33(6):1011–9. doi: 10.1038/s41594-026-01821-8 (PMC13275289; doi:10.1038/s41594-026-01821-8)

Extended Data Figure 4b

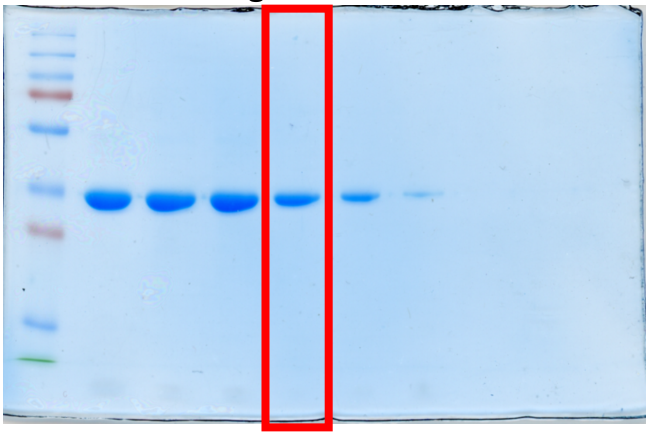

Extended Data Figure 4c

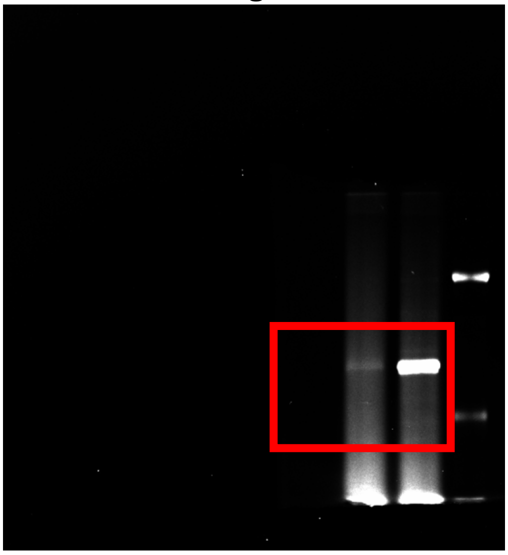

Extended Data Figure 4d

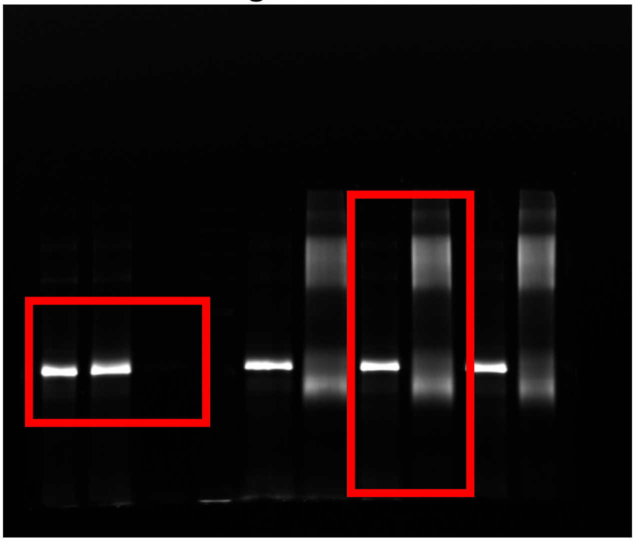

Extended Data Figure 6a

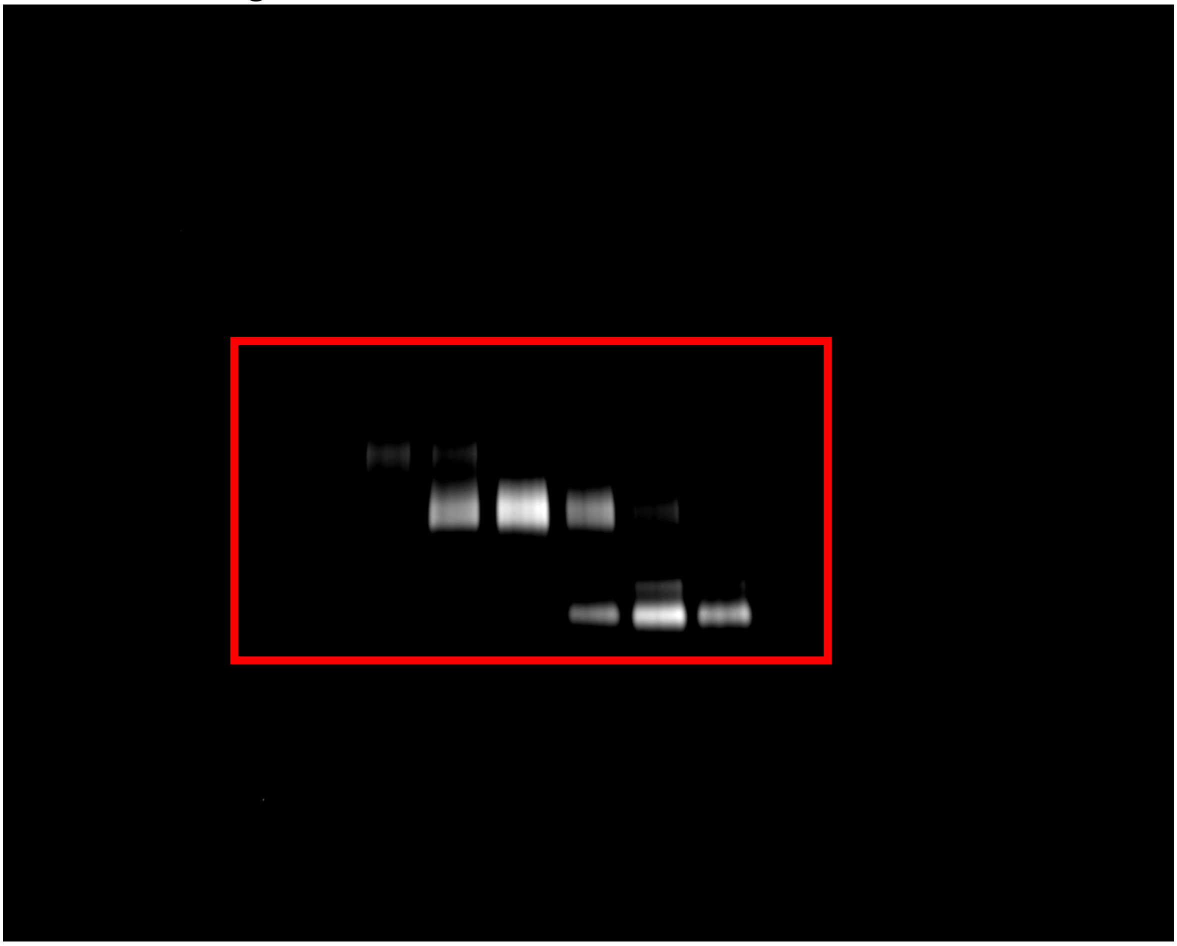

Extended Data Figure 9a

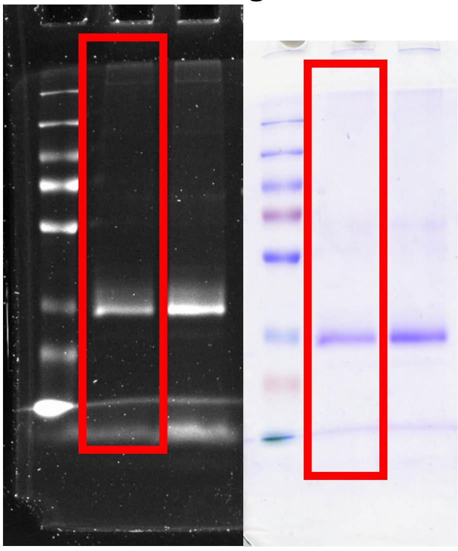

Supplement: Supplementary file 6 — Uncropped gels. [file 41594_2026_1821_MOESM6_ESM.pdf]
