# Supplementary material for: A single-vesicle fluorescence microscopy platform to quantify phospholipid scrambling
Source: Nat Struct Mol Biol. 2026 Jun 15;33(6):1011–9. doi: 10.1038/s41594-026-01821-8 (PMC13275289; doi:10.1038/s41594-026-01821-8)
Supplement: Supplementary file 3 — Peer Review File [file 41594_2026_1821_MOESM3_ESM.pdf]

nature portfolio

Peer Review File

A single vesicle fluorescence microscopy platform to quantify phospholipid scrambling

Corresponding Author: Professor Anant Menon

Version 0:

Decision Letter:

30th Jun 2025

Dear Anant,

I am writing on Kat's behalf as she is out of the office. I am deeply sorry for the delay in sharing our decision with you.

Thank you again for submitting your manuscript "A single vesicle fluorescence microscopy platform to quantify phospholipid scrambling". I apologize for the delay in responding again. We now have comments (below) from the 3 reviewers who evaluated your paper. In light of those reports, we remain interested in your study and would like to see your response to the comments of the referees, in the form of a revised manuscript.

You will see that the reviewers were supportive of the technical advance, but also asked for additional characterizations and test of the breadth of applicability and limits of the method. We have discussed their points editorially and agree that it will be important to more fully characterize the method (in terms of effect of temperature, Rev#2 pt #3 and applicability to other proteins, Rev#2 pt #2, and we also agree with revising to show this method can be extended to other lipid species, Rev#2 pt #1). Please also address the concerns of Rev#3 about the kinetic modelling analyses and Rev#1's suggestions for the presentation of the method, what it requires, and what its limits are. Please address the referees' minor points and requests to strengthen existing evidence.

We are committed to providing a fair and constructive peer-review process and our standard revision process is 6 months. Please do not hesitate to contact us if there are specific requests from the reviewers that you believe are technically impossible or unlikely to yield a meaningful outcome. If you cannot send your revision within this time, please let us know. We will be happy to consider your revision as long as nothing similar has been accepted for publication at NSMB or published elsewhere. Should your manuscript be substantially delayed without notifying us in advance and your article is eventually published, the received date would be that of the revised, not the original, version.

As you already know, we put great emphasis on ensuring that the methods and statistics reported in our papers are correct and accurate. As such, if there are any changes that should be reported, please submit an updated version of the Reporting Summary along with your revision.

Reporting Summary:
https://www.nature.com/documents/nr-reporting-summary.pdf

Please note that the form is a dynamic ‘smart pdf’ and must therefore be downloaded and completed in Adobe Reader.

When submitting the revised version of your manuscript, please pay close attention to our href="https://www.nature.com/nature-portfolio/editorial-policies/image-integrity">Digital Image Integrity Guidelines. and to the following points below:

-- that unprocessed scans are clearly labelled and match the gels and western blots presented in figures. Please note that all key data shown in the main figures as cropped gels or blots should be presented in uncropped form, with molecular weight markers. While these data can be displayed in a relatively informal style, they must refer back to the relevant figures. These data should be submitted as source data with the last revision, prior to acceptance.
-- that control panels for gels and western blots are appropriately described as loading on sample processing controls
-- all images in the paper are checked for duplication of panels and for splicing of gel lanes.
-- For any revision that includes light microscopy data, we ask our authors to please include a completed light microscopy reporting table [https://www.nature.com/documents/Light_microscopy_reporting_table.xlsx] to ensure the methods are

described thoroughly. The table will be available to reviewers and ultimately published should the manuscript be accepted at the journal.

EXTENDED DATA FIGURES

When re-submitting your manuscript, please ensure that any supplementary figures and tables that are crucial to the manuscript's conclusions are converted into Extended Data figures and tables to increase visibility of these data. Extended Data figures and tables are online-only (present in the online PDF and full-text HTML versions of the paper), peer-reviewed display items that provide essential background to the article but are not included in the main article due to space constraints. A maximum of ten Extended Data display items (figures and tables) is permitted.

Finally, please ensure that you retain unprocessed data and metadata files after publication, ideally archiving data in perpetuity, as these may be requested during the peer review and production process or after publication if any issues arise.

If there are additional or modified structures presented in the final revision, please submit the corresponding PDB validation reports.

SOURCE DATA: we urge authors to provide, in tabular form, the data underlying the graphical representations used in figures. This is to further increase transparency in data reporting, as detailed in this editorial (http://www.nature.com/nsmb/journal/v22/n10/full/nsmb.3110.html). Spreadsheets can be submitted in excel format. Only one (1) file per figure is permitted; thus, for multi-paneled figures, the source data for each panel should be clearly labeled in the Excel file; alternately the data can be provided as multiple, clearly labeled sheets in an Excel file. When submitting files, the title field should indicate which figure the source data pertains to. We encourage our authors to provide source data at the revision stage, so that they are part of the peer-review process.

While we encourage the use of color in preparing figures, please note that this will incur a charge to partially defray the cost of printing. Information about color charges can be found at http://www.nature.com/nsmb/authors/submit/index.html#costs

We require deposition of coordinates (and, in the case of crystal structures, structure factors) into the Protein Data Bank with the designation of immediate release upon publication (HPUB). Electron microscopy-derived density maps and coordinate data must be deposited in EMDB and released upon publication. Deposition and immediate release of NMR chemical shift assignments are highly encouraged. Deposition of deep sequencing and microarray data is mandatory, and the datasets must be released prior to or upon publication. To avoid delays in publication, dataset accession numbers must be supplied with the final accepted manuscript and appropriate release dates must be indicated at the galley proof stage. Please find the complete NRG policies on data availability at http://www.nature.com/authors/policies/availability.html.

Nature Structural & Molecular Biology is committed to improving transparency in authorship. As part of our efforts in this direction, we are now requesting that all authors identified as 'corresponding author' on published papers create and link their Open Researcher and Contributor Identifier (ORCID) with their account on the Manuscript Tracking System (MTS), prior to acceptance. This applies to primary research papers only. ORCID helps the scientific community achieve unambiguous attribution of all scholarly contributions. You can create and link your ORCID from the home page of the MTS by clicking on 'Modify my Springer Nature account'. For more information please visit please visit www.springernature.com/orcid.

Please use the link below to submit your revised manuscript and related files:

Link Redacted

Note: This URL links to your confidential home page and associated information about manuscripts you may have submitted, or that you are reviewing for us. If you wish to forward this email to co-authors, please delete the link to your homepage.

We look forward to seeing the revised manuscript and thank you for the opportunity to review your work.

Sincerely,

Melina Casadio, PhD
Locum Chief Editor, Nature Structural & Molecular Biology
ORCID ID: https://orcid.org/0000-0003-2389-2243

Referee expertise:

Referee #1: imaging

Referee #2: membrane biology, lipids

Referee #3: lipid transport

Reviewers' Comments:

Reviewer #1 (Remarks to the Author):

The authors present the design and realization of an assay to determine lipid scrambling activity of the scramblase VDAC1 on a single vesicle level using fluorescence microscopy in combination with a fluorescent lipid analogue and a flow chamber. Scramblases are important since they ensure lipid exchange between inner and outer membrane leaflets and thus lipid transport across membranes. The center-piece is the use of a fluorescent lipid analogue that can desorb to a small fraction from the outer leaflet, which thus leads to a decrease of fluorescence per vesicle and a total loss of fluorescence only upon active scrambling of the lipid analogues from the inner leaflet. The authors exemplify this assay for the scramblase VDAC1, which is only actively scrambling when dimerized. Following this, the authors highlight large heterogeneity in scrambling activity, which is otherwise hidden in ensemble measurements.

This is a very nice and well conducted and written story. It is well motivated, excellent controls are conducted and all necessary details are given. I would in principle like to see this published.

Apart from a few minor remarks (see below), I however ask myself, whether this story is of high enough impact for Nature Structural & Molecular Biology. It is as highlighted very well performed research with excellent results. However, the basic idea of using the fluorescent lipid analogue is not novel and the whole design thus not outstandingly new. Still I would like to give it a chance. Maybe the authors could detail more on how readily implementable this assay platform really is, including a more detailed guideline on how to build it up technically (flow chamber, microscope, analysis) as well as what biochemical issues might come up (reconstitution is still needed)?

Minor comments:
- Abstract: I would name here already what VDAC1 is (a channel, scramblase).
- One could change the lipid composition – what changes could one expect? The authors could add some discussion on this already in the main text and mention what lipid composition has been chosen and why.
- I suggest not to use wordings like quenching or bleaching for loss in fluorescence signal upon desorption of the lipid analogue. Maybe just name it fluorescence loss.
- Page 8, line 194ff: Detail on the dependencies on the flow rate and why exactly one rate has been chosen.
- Line 254: Why 45% here? Did not get this.
- Briefly state somewhere in the main text how the ensemble assay was conducted.
- Can the cross-linker have any other influence on VDAC1 then just dimerization – e.g. change in channel activity?
- Figure 3c: The correlation between protein content and signal reduction could maybe be highlighted more detailed (e.g. via a two-dimensional scatter plot)?

Reviewer #1 (Remarks on code availability):

I did not receive any code. Seems to be only upon request.

Reviewer #2 (Remarks to the Author):

Phospholipid scramblases play critical roles in numerous physiological processes by facilitating the bidirectional movement of lipids across biological membranes. In this study, Vei et al. present a compelling single-vesicle analysis platform that enables quantitative assessment of lipid scrambling at the level of individual vesicles. By using fluorescently labeled phospholipids and vesicles reconstituted with heterogeneously crosslinked VDAC1 dimers, the authors demonstrate that this approach allows for concurrent measurement of vesicle size, protein incorporation, and lipid transport rates. Notably, this method reveals a striking heterogeneity in scrambling activity among individual VDAC1 dimers, ranging from fewer than 100 to over 10,000 lipids per second, a degree of kinetic diversity that would be ignored in traditional bulk assays. This platform represents a significant technical advance, providing a powerful means to investigate the functional dynamics and regulation of scramblases with unprecedented resolution.

I believe the analytical framework introduced in this study is highly innovative and has the potential to broadly impact the field of membrane biology. To fully demonstrate its capabilities and support its broad applicability, I recommend addressing the following three points. Clarification of these aspects would substantially strengthen the manuscript and enhance its suitability for publication in a top journal such as Nature Structural & Molecular Biology:

1.Applicability to Diverse Lipid Species
Can the platform be used to study lipid scrambling of phospholipids beyond phosphatidylcholine (PC), such as phosphatidylserine (PS) or sphingomyelin (SM)? If so, the authors should consider comparing the desorption or scrambling kinetics of these lipids relative to PC. Such data would not only underscore the system's versatility but also allow investigation of lipid selectivity or preferences of different scramblases. In particular, SM may be advantageous due to its typically slower scrambling rate, which could facilitate comparative analysis with PC, having the same head group phosphocholine.

2.Sensitivity to other Scramblases

Can this system capture lipid scrambling by proteins with faster transport rates than VDAC1, such as members of the TMEM16 family? If there is a technical upper limit in rate detection, could it be extended—for example, by modulating vesicle size or lipid composition? Establishing the system's dynamic range would be crucial for defining its scope of applicability. Additionally, in line 432, the authors compare the VDAC1 kinetics obtained here with prior reports on TMEM16. Direct measurements of TMEM16 using the same assay platform would allow for a more meaningful comparison and strengthen the discussion (e.g., line 435). TMEM16 is also a favorable test case given its calcium-regulated activity, which could be used to test dynamic changes in scrambling under controlled conditions.

3.Temperature Sensitivity
To what extent is the assay sensitive to temperature variations? While the scrambling rate itself is expected to be temperature-dependent, it would be helpful to know whether background activity—such as that from empty liposomes or non-crosslinked VDAC1—also varies with temperature. Understanding this would improve interpretability of the data and help establish optimal experimental parameters for future applications.

Reviewer #3 (Remarks to the Author):

Veit et al. report a single-vesicle assay for quantitatively measuring the kinetics protein-catalyzed lipid scrambling by single scramblase complexes. They reconstitute VDAC1-dimers in proteoliposomes and use a precise chemical crosslinking strategy to mimic the high VDAC1 concentration in the outer mitochondrial membrane, which would otherwise be difficult to recapitulate. These proteoliposomes are anchored on a solid support, and spiked with NBD lipids, either ion a symmetric or asymmetric distribution. Through a rather sophisticated combination of TIRF microscopy, lipid extraction through albumin excess and photobleaching, the authors are able to obtain kinetic traces from individual vesicles that contain only a single active scramblase complex.
These datasets – which I have not seen in such high quality – allow them to estimate the scrambling rates and amount of lipids scrambled per protein over time through kinetic modelling.
This high-throughput microscopy assay represents a significant step forward and is also a very timely contribution. There is quite a bit of evidence by now that lipid transbilayer movement is closely linked to inter-organelle lipid transport and thus the fundamental question of organelle membrane identity. Putting hard numbers on the rates of lipid transbilayer movement has never been important than today and the present manuscript goes a long way towards this goal. Therefore, I am in favor of publishing this manuscript in NSMB.
Regarding specifics with regard to this manuscript, I have no issues with the biochemistry, the experimental design, which I like quite a lot, very cool biochemistry/quantitative microscopy. I do have some concerns, however, with regard to the kinetic modelling, where I am worried about a potential overfitting effect. This can be tested in a reasonably straightforward manner with little additional experimental effort.

Model fitting comment:

The kinetic model makes sense, and it may very well be that it is wholly appropriate to fit the data as the authors have done. I would, however would like to see a thorough testing of model choice and number of free parameters as I worry about the following:
In Figure 3c (single traces) and f (category averages) it appears that the single vesicle kinetic traces sort pretty much according to protein content which varies by a little more than one order of magnitude (if I see it correctly between 0 and 15 dimers per vesicle). The derived scrambling parameter alpha in contrast varies by three orders of magnitude. Taken these two things together, I would intuitively expect the following:

1. A very loose ordering of kinetic traces by protein concentration – much less pronounced to what was observed.

2. Higher standard deviation for kinetic traces derived from low-protein content vesicles compared to high protein-content vesicles, in particular for early timepoints. The former population captures the full activity distribution of very active and almost inactive dimers, whereas the latter have a much higher probability of containing at least one highly active dimer, which should then dominate the scrambling process. As far as I can see this trend is not observed.

There may very well be a reason that I am not aware off that may explain these observations, but the worry is always that the parameters become difficult to identify when there is not sufficient information in the kinetic traces (as is often the case with monotonously declining, noisy traces). It is possible that three free (but constrained) parameters allow for multiple combinations of parameter values that give a reasonable fit which could widen the observed distribution of values for alpha, in particular considering that these are single vesicle measurements, which are inherently noisy.

I would suggest the following:

1. I suggest deriving gamma from the asymmetric tail-labelled NBD PC population (if necessary, using a new dataset with higher time resolution) and using this as a fixed value in a model re-run for the traces contributing to h-j. These two models can then be compared using Akaike's information criterion or related approaches.

2. Quite a few "single dimer traces" do not appear to fall outside of the NBD F/F0 range between 0.4 and 0.6 which is observed for empty vesicles, where scrambling should not take place. In these cases, I would argue that it is unclear whether

any scrambling takes place in the respective vesicle subpopulation of "single dimer" vesicles. How would model fits of this subset of traces compare to fits of the empty traces? To which extent would the distribution of alpha change if these traces were excluded? Related: It is possible that the initial constraint of the Lout/(Lin+Lout) ratio of 45-55% forces the model into scrambling when there is probably none, as this is less wide as the range actually observed for “empty” vesicles.

3. Another possibility would be a bootstrapping approach – the authors could use subsets of kinetic traces to generate less noisy traces and gauge the variability in the kinetic parameters in this way.

I do believe that these re-analyses would make the paper much stronger – and since this is the best dataset on lipid scrambling activity that I am aware of, it will likely constitute a reference point for the literature going forward. Against that background I think that it is advisable to have a well-validated model choice in the paper.

Minor comments

Figure 1d: The shape of theoretical traces does not look exponential – I’d expect an exponential decay of some sort, not a mostly linear function

I recommend to not use SDT as an abbreviation for sodium dithionite. The authors can use the chemical formula if they think an abbreviation is necessary (which may be needed in the figure), but I do not think that establishing yet another custom abbreviation helps the readability.

Figure legend 2d: “This was followed by sodium dithionite addition to selectively bleach inner-leaflet NBD lipids”. I understand what the authors are trying to say here, but as a standalone sentence it is misleading.

Figure 2e, Figure 3: I recommend not inverting the typical colormap of the fire lookup table – yellow usually encodes the highest values while violet encodes low values

André Nadler

Version 1:

Decision Letter:

Our ref: NSMB-TR50979A

5th Feb 2026

Dear Dr. Menon,

Thank you for submitting your revised manuscript "A single vesicle fluorescence microscopy platform to quantify phospholipid scrambling" (NSMB-TR50979A). It has now been seen by the original referees and their comments are below. The reviewers find that the paper has improved in revision, and therefore we'll be happy in principle to publish it in Nature Structural & Molecular Biology, pending minor revisions to satisfy the referees' final requests and to comply with our editorial and formatting guidelines. Specifically, we will request that comments of reviewer 2 regarding VDAC1 and opsin scrambling activity are addressed textually.

We are now performing detailed checks on your paper and will send you a checklist detailing our editorial and formatting requirements in about 2-3 weeks. Please do not upload the final materials and make any revisions until you receive this additional information from us.

To facilitate our work at this stage, it is important that we have a copy of the main text as a word file. If you could please send along a word version of this file as soon as possible, we would greatly appreciate it; please make sure to copy the NSMB account (cc'ed above).

Thank you again for your interest in Nature Structural & Molecular Biology Please do not hesitate to contact me if you have any questions.

Sincerely,

Katarzyna Ciazynska, PhD
(she/her)
Senior Editor
Nature Structural & Molecular Biology
https://orcid.org/0000-0002-9899-2428

Reviewer #1 (Remarks to the Author):

The authors have very well commented to all of my concerns and excellently revised the manuscript (especially through the

additional experiments, data and figures). I suggest publication of this nice work.

Reviewer #2 (Remarks to the Author):

The authors have addressed most of the points we previously raised.

With respect to the reconstitution experiments, the authors chose opsin rather than TMEM16. However, the lipid-scrambling activity of VDAC1 and opsin has so far been evaluated primarily in reconstitution systems and has not been clearly demonstrated at the cellular level unlike TMEM16.

Although opsin is thought to be constitutively active, its lipid-scrambling activity has not, to our knowledge, been convincingly demonstrated in cells. Therefore, some uncertainty remains regarding the rationale for using opsin instead of TMEM16 (which is Ca^{2+}-stimulated but not constitutively active) as a reference scramblase.

Nevertheless, given that this work presents a platform technology for observing lipid scrambling at the single-vesicle level in cells, this reviewer believes that the revision has been carried out successfully.

Reviewer #3 (Remarks to the Author):

The authors sufficiently addressed my comments in their revisions and I now recommend acceptance w/o any further revisions. Congratulations to a study well done!

P.S. I still think that inverting a very common color scheme (Fire Lut) is a debatable data visualization choice, but since this is a matter of taste/design, I shall not press the point.

Version 2:

Decision Letter:

5th May 2026

Dear Dr. Menon,

We are now happy to accept your revised paper "A single vesicle fluorescence microscopy platform to quantify phospholipid scrambling" for publication as a Technical Report in Nature Structural & Molecular Biology.

Acceptance is conditional on the manuscript's not being published elsewhere and on there being no announcement of this work to the newspapers, magazines, radio or television until the publication date in Nature Structural & Molecular Biology.

Over the next few weeks, your paper will be copyedited to ensure that it conforms to Nature Structural & Molecular Biology style. Once your paper is typeset, you will receive an email with a link to choose the appropriate publishing options for your paper and our Author Services team will be in touch regarding any additional information that may be required.

After the grant of rights is completed, you will receive a link to your electronic proof via email with a request to make any corrections within 48 hours. If, when you receive your proof, you cannot meet this deadline, please inform us at rjsproduction@springernature.com immediately.

You will not receive your proofs until the publishing agreement has been received through our system.

Due to the importance of these deadlines, we ask that you please let us know now whether you will be difficult to contact over the next month. If this is the case, we ask you provide us with the contact information (email, phone and fax) of someone who will be able to check the proofs on your behalf, and who will be available to address any last-minute problems.

To assist our authors in disseminating their research to the broader community, our SharedIt initiative provides all co-authors with the ability to generate a unique shareable link that will allow anyone (with or without a subscription) to read the published article. Recipients of the link with a subscription will also be able to download and print the PDF.

As soon as your article is published, you can generate your shareable link by entering the DOI of your article here: http://authors.springernature.com/share. Corresponding authors will also

receive an automated email with the shareable link

Note the policy of the journal on data deposition: http://www.nature.com/authors/policies/availability.html.

Your paper will be published online soon after we receive proof corrections and will appear in print in the next available issue. You can find out your date of online publication by contacting the production team shortly after sending your proof corrections.

You may wish to make your media relations office aware of your accepted publication, in case they consider it appropriate to organize some internal or external publicity. Once your paper has been scheduled you will receive an email confirming the publication details. This is normally 3-4 working days in advance of publication. If you need additional notice of the date and time of publication, please let the production team know when you receive the proof of your article to ensure there is sufficient time to coordinate. Further information on our embargo policies can be found here: https://www.nature.com/authors/policies/embargo.html

You can now use a single sign-on for all your accounts, view the status of all your manuscript submissions and reviews, access usage statistics for your published articles and download a record of your refereeing activity for the Nature journals.

If you have not already done so, we strongly recommend that you upload the step-by-step protocols used in this manuscript to the Protocol Exchange. Protocol Exchange is an open online resource that allows researchers to share their detailed experimental know-how. All uploaded protocols are made freely available, assigned DOIs for ease of citation and fully searchable through nature.com. Protocols can be linked to any publications in which they are used and will be linked to from your article. You can also establish a dedicated page to collect all your lab Protocols. By uploading your Protocols to Protocol Exchange, you are enabling researchers to more readily reproduce or adapt the methodology you use, as well as increasing the visibility of your protocols and papers. Upload your Protocols at www.nature.com/protocolexchange/. Further information can be found at www.nature.com/protocolexchange/about.

An online order form for reprints of your paper is available at https://www.nature.com/reprints/author-reprints.html. Please let your coauthors and your institutions' public affairs office know that they are also welcome to order reprints by this method.

Authors may need to take specific actions to achieve compliance with funder and institutional open access mandates. If your research is supported by a funder that requires immediate open access (e.g. according to Plan S principles or the NIH public access policy) then you should select the gold OA route, and we will direct you to the compliant route where possible. Because authors warrant under our subscription licensing terms that they haven't committed to licensing any version of their article under a licence inconsistent with the terms of our agreement – including the applicable embargo period – publication under the subscription model isn't suitable for authors whose funders require no embargo.

In approximately 10 business days you will receive an email with a link to choose the appropriate publishing options for your paper and our Author Services team will be in touch regarding any additional information that may be required.

You will not receive your proofs until the publishing agreement has been received through our system.

If you have any questions about our publishing options, costs, Open Access requirements, or our legal forms, please contact ASJournals@springernature.com

Sincerely,

Katarzyna Ciazynska, PhD
(she/her)
Senior Editor
Nature Structural & Molecular Biology
https://orcid.org/0000-0002-9899-2428

Click here if you would like to recommend Nature Structural & Molecular Biology to your librarian: http://www.nature.com/subscriptions/recommend.html#forms

Open Access This Peer Review File is licensed under a Creative Commons Attribution 4.0 International License, which permits use, sharing, adaptation, distribution and reproduction in any medium or format, as long as you give appropriate credit to the original author(s) and the source, provide a link to the Creative Commons license, and indicate if changes were made.
In cases where reviewers are anonymous, credit should be given to 'Anonymous Referee' and the source.
The images or other third party material in this Peer Review File are included in the article's Creative Commons license, unless indicated otherwise in a credit line to the material. If material is not included in the article's Creative Commons license and your intended use is not permitted by statutory regulation or exceeds the permitted use, you will need to obtain permission directly from the copyright holder.
To view a copy of this license, visit https://creativecommons.org/licenses/by/4.0/

NSMB-TR50979
Veit et al.
A single vesicle fluorescence microscopy platform to quantify phospholipid scrambling

Response to editorial summary and reviewer's comments

Editorial summary:
We have discussed their points editorially and agree that it will be important to more fully characterize the method (in terms of effect of temperature, Rev#2 pt #3 and applicability to other proteins, Rev#2 pt #2, and we also agree with revising to show this method can be extended to other lipid species, Rev#2 pt #1). Please also address the concerns of Rev#3 about the kinetic modelling analyses and Rev#1's suggestions for the presentation of the method, what it requires, and what its limits are.

The following is an overview of our response to the editorial summary; details are presented within our responses to the comments of the individual reviewers:
1. Effect of temperature – the effect of temperature on the desorption rate constant is presented in new Extended Data Fig. 2g and described in the Results (line 215ff)
2. Applicability to other proteins – data on the GPCR scramblase opsin are presented in new Extended Data Figure 8, Methods (line 660ff) and Results (line 453ff)
3. Extension to other lipid species – results for a suite of fluorescent reporter lipids are presented in new Extended Data Fig. 2h and described in the Results (line 219ff)
4. Concerns about kinetic modeling analyses – these are addressed in the Results (line 497ff) and Methods (line 740ff) sections and in new Extended Data Table 1
5. Presentation of the single vesicle method and its requirements and limits – these points are addressed in the new Extended Data Figure 1, Results line 171ff, Discussion (line 525ff and line 541ff) and throughout the text where the method is discussed.

Reviewer #1 (Remarks to the Author):

The authors present the design and realization of an assay to determine lipid scrambling activity of the scramblase VDAC1 on a single vesicle level using fluorescence microscopy in combination with a fluorescent lipid analogue and a flow chamber. Scramblases are important since they ensure lipid exchange between inner and outer membrane leaflets and thus lipid transport across membranes. The center-piece is the use of a fluorescent lipid analogue that can desorb to a small fraction from the outer leaflet, which thus leads to a decrease of fluorescence per vesicle and a total loss of fluorescence only upon active scrambling of the lipid analogues from the inner leaflet. The authors exemplify this assay for the scramblase VDAC1, which is only actively scrambling when dimerized. Following this, the authors highlight large heterogeneity in scrambling activity, which is otherwise hidden in ensemble measurements.

This is a very nice and well conducted and written story. It is well motivated, excellent controls are conducted and all necessary details are given. I would in principle like to see this published.

Apart from a few minor remarks (see below), I however ask myself, whether this story is of high enough impact for Nature Structural & Molecular Biology. It is as highlighted very well performed research with excellent results. However, the basic idea of using the fluorescent lipid analogue is not novel and the whole design thus not outstandingly new. Still I would like to give it a chance. Maybe the authors could detail more on how readily implementable this assay platform really is, including a more detailed guideline

on how to build it up technically (flow chamber, microscope, analysis) as well as what biochemical issues might come up (reconstitution is still needed)?

We greatly appreciate the reviewer's positive comments and address the general issues raised as follows:

"the basic idea of using the fluorescent lipid analogue is not novel and the whole design thus not outstandingly new" Fluorescent lipid analogs have long been used in the study of intracellular lipid transport and specifically transbilayer lipid movement. They are the reporters of choice for activity assays of reconstituted scramblases. However, such assays are invariably performed at the ensemble level, with associated limitations as described in the Introduction (line 73ff). Our work overcomes these constraints by measuring the activity of a single scramblase within a single vesicle containing a defined number of phospholipids. This is without precedent in the field, and we believe it to be a major step forward. We note that even though we use NBD lipids, the core principle of our assay, which requires their desorption and subsequent washout, is technically new and has not been previously described.

"how readily implementable this assay platform really is, including a more detailed guideline on how to build it up technically (flow chamber, microscope, analysis)" In response to the reviewer's question regarding the assay setup, we have now included a new figure (Extended Data Figure 1) that provides stepwise schematics and photographs illustrating how the sample chamber is prepared, assembled and connected to the peristaltic pump which is used for introducing the sample and providing the buffer flow to remove desorbed NBD lipids. This figure highlights the relative simplicity of the assay setup and shows how it can be implemented. Data analysis requires access to software which, as stated at the end of the manuscript, is available upon request.

"as well as what biochemical issues might come up (reconstitution is still needed)?" To measure the activity of a single scramblase, it is necessary to reconstitute a fluorescently-labeled variant of the protein. We described this process for both VDAC1 and now add descriptions for the GPCR opsin (new Extended Data Figure 8, and associated Methods (line 660ff) and Results (line 453ff)). The proteins were labeled with Alexa Fluor 647 at 1:1 stoichiometry and reconstituted into large unilamellar vesicles, where they exhibited the expected scramblase activity in ensemble assays, e.g., Extended Data Figure 5c (VDAC1) and. Extended Data Figure 8c,d (opsin). These samples were then taken for single protein/single vesicle measurements. Thus, stoichiometric fluorescence labeling of a purified scramblase and its reconstitution into unilamellar vesicles is a prerequisite for our analyses. This is implied in many places in the text, stated explicitly on line 171ff and restated at the start of the discussion (line 475ff). For typical ensemble assays that are used generally in the field, fluorescence labeling of the scramblase is not needed, but the requirement for purified protein and vesicle reconstitution remains.

Minor comments:
- Abstract: I would name here already what VDAC1 is (a channel, scramblase).
We have spelled out VDAC1 in the abstract.

- One could change the lipid composition – what changes could one expect? The authors could add some discussion on this already in the main text and mention what lipid composition has been chosen and why.
We added a comment on the choice of lipid composition in the Discussion as follows (line 515ff): 'We note that in the experiments reported here we used a simple fluid membrane matrix for reconstitution, comprising POPC with 10 mole percentage POPG. This mixture is widely used as it supports the activity of most scramblases. Heterogeneity within a liposome preparation made up of a complex lipid mixture precludes the ready dissection of composition-specific effects in ensemble assays – such analyses are now possible in our single vesicle platform where individual scramblase-containing vesicles can be

characterized by properties such as fluidity, or directly in terms of their lipid makeup. Thus, it will now be possible to test precisely the effect of different lipids, including cholesterol, on scramblase activity.'

We note that it is difficult to predict the of lipid composition changes on scramblase activity as these could occur via changes in the physical properties of the membrane, for example bilayer thickness or fluidity, and/or via the effect of specific lipids that bind to the scramblase to act as allosteric effectors. For example, in recent work on the effect of cholesterol on GPCR scramblases we found that cholesterol acts as a switch, blocking scramblase activity above a certain threshold (Menon et al. (2024) J Biol Chem, cited as reference 17). As noted above, our single vesicle approach provides an excellent platform to investigate these points in follow-up studies.

- I suggest not to use wordings like quenching or bleaching for loss in fluorescence signal upon desorption of the lipid analogue. Maybe just name it fluorescence loss.

Thank you for this comment. We have now gone through the manuscript to make sure that we have used these terms precisely. For convenience, a glossary is provided below.

Bleaching refers to the irreversible loss of fluorescence which occurs, for example, when dithionite reacts with the NBD fluorophore and converts it to a non-fluorescent derivative. This term applies to our single-vesicle dithionite assay (Fig. 2d-f, Extended Data Fig. 3e, Extended Data Fig. 4, Extended Data Fig. 5b, Extended Data Fig. 8a).

Quenching refers to the partial loss of fluorescence which occurs when desorbed NBD lipids are captured by albumin (the quantum efficiency of NBD when the NBD lipid is bound to albumin is lower than when the lipid is in the membrane).

In our flow scramblase assay set up, neither term applies as desorbed NBD lipids are washed away, and therefore their fluorescence is lost.

- Page 8, line 194ff: Detail on the dependencies on the flow rate and why exactly one rate has been chosen.

We now explain that a flow rate of 500 µL/min was chosen to balance the need to have fast removal of desorbed NBD-lipid versus the need to retain image focus and microscope stability (line 214ff). In new Extended Data Figure 2f, we now provide additional results showing the effect of varying the flow rate on the extraction rate constant γ.

- Line 254: Why 45% here? Did not get this.

Thank you for raising this point as our original text was not sufficiently clear. This is now explained in detail in the Results (line 298ff) and figures (Fig. 2e, Extended Data Figure 4a,b) as follows: ' In a very small subset of vesicles, we found that as much as ~55% of the fluorescence was lost, likely because NBD-PC molecules in the outer leaflet of these vesicles had not been completely removed during the initial phase of the experiment (Fig. 2e, first column, Extended Data Fig. 4a). Accordingly, we chose 45% residual fluorescence as a cut-off (Extended Data Fig. 4a,b, dashed line indicates cut-off) to distinguish protein-free vesicles from those containing VDAC1 channels which permit $[S_2O_4]^{2-}$ entry, and which consequently lose all fluorescence on dithionite treatment (Fig. 2e).'

A longer explanation of this choice is as follows. We introduce protein-free vesicles into the sample chamber over a 1-min period, and allow them to immobilize for a further 1-min. Then we initiate buffer flow for 5 min to eliminate all outer leaflet NBD-PC molecules (these desorb from the outer leaflet and are washed away by buffer). Then 3 images are taken, the average of these is termed F_0. Following image acquisition, samples are mock-treated for several minutes and we observe a small fluorescence drop (~8% on average, based on almost 1,500 vesicles measured, represented by the average values – black symbols – plotted in Extended Data Figure 4a) likely due to a combination of bleaching, microscope stage movement because of multi-position imaging, and loss of residual NBD-PC from the outer leaflet. This is a

minor effect, but for a small subset of vesicles greater fluorescence changes are observed (line 298ff), a result of the statistical nature of the outcome of single vesicle experiments. To discriminate clearly between protein-free vesicles and VDAC1-channel containing vesicles, we use a cut-off of 0.45 to eliminate these outliers. For clarification, we added a horizontal line with the 0.45 cutoff to Figure 2e and Extended Data Figure 4a, b. The data showing two pools of vesicles (top half in yellow, bottom half in stronger colors indicative of protein) make the 0.45 cut-off evident in these figures.

- Briefly state somewhere in the main text how the ensemble assay was conducted.
This is now described in the Methods section (line 647ff).

- Can the cross-linker have any other influence on VDAC1 then just dimerization – e.g. change in channel activity?
The crosslinker has no discernible effect on channel activity as evaluated by dithionite permeation (Extended Data Fig. 3e, compare red (CL) and dark green (NCL) traces corresponding to crosslinked (CL) and non-crosslinked (NCL) VDAC1). However as noted previously (Jahn et al. (2023) Nature Commun, cited as reference 12), *intra*molecular crosslinking results in compaction of the VDAC1 structure such that it migrates faster on SDS-PAGE (Extended Data Fig. 3d, the lower monomer band in the CL lane migrates faster than its counterpart in the NCL lane, as noted on lines 976-977 in the figure legend).

- Figure 3c: The correlation between protein content and signal reduction could maybe be highlighted more detailed (e.g. via a two-dimensional scatter plot)?
We considered this suggestion carefully and, in the end, thought that the conversion of the data in panel **c** to the category averaged format in panel **f** offered a simplification that would make it easy for a reader to capture the result. The ultimate goal is to identify vesicles with a single VDAC1 dimer, i.e., vesicles that show two-step Alexa Fluor 647 photobleaching (as seen in panel **h**, and Extended Data Figure 7), and these are buried amongst the numerous traces shown in panel **c**. Thus, we have left the figure as is.

Reviewer #1 (Remarks on code availability):

I did not receive any code. Seems to be only upon request.
The code was provided with the original submission as required by NSMB. We regret that it was not available to the reviewer.

Reviewer #2 (Remarks to the Author):

Phospholipid scramblases play critical roles in numerous physiological processes by facilitating the bidirectional movement of lipids across biological membranes. In this study, Vei et al. present a compelling single-vesicle analysis platform that enables quantitative assessment of lipid scrambling at the level of individual vesicles. By using fluorescently labeled phospholipids and vesicles reconstituted with heterogeneously crosslinked VDAC1 dimers, the authors demonstrate that this approach allows for concurrent measurement of vesicle size, protein incorporation, and lipid transport rates. Notably, this method reveals a striking heterogeneity in scrambling activity among individual VDAC1 dimers, ranging from fewer than 100 to over 10,000 lipids per second, a degree of kinetic diversity that would be ignored in traditional bulk assays. This platform represents a significant technical advance, providing a powerful means to investigate the functional dynamics and regulation of scramblases with unprecedented resolution.

I believe the analytical framework introduced in this study is highly innovative and has the potential to

broadly impact the field of membrane biology. To fully demonstrate its capabilities and support its broad applicability, I recommend addressing the following three points. Clarification of these aspects would substantially strengthen the manuscript and enhance its suitability for publication in a top journal such as Nature Structural & Molecular Biology:

We appreciate the reviewer's positive assessment of our manuscript. Our response to the reviewer's specific points is detailed below.

1.Applicability to Diverse Lipid Species
Can the platform be used to study lipid scrambling of phospholipids beyond phosphatidylcholine (PC), such as phosphatidylserine (PS) or sphingomyelin (SM)? If so, the authors should consider comparing the desorption or scrambling kinetics of these lipids relative to PC. Such data would not only underscore the system's versatility but also allow investigation of lipid selectivity or preferences of different scramblases. In particular, SM may be advantageous due to its typically slower scrambling rate, which could facilitate comparative analysis with PC, having the same head group phosphocholine.
Thank you for this suggestion. We have now examined the suitability of various NBD-labeled reporter lipids for this assay by evaluating their desorption rate constant (γ) under our standard temperature and flow rate conditions. We show that an increase in the 1-acyl chain length predictably decreases γ, but C6-sphingomyelin, on account of the enhanced polarity resulting from the 3-OH on its sphingosine backbone, has a higher γ than the corresponding PC. These data are presented in new Extended Data Figure 2h and discussed in line 219ff . Thus, the platform can be indeed used for studying the scrambling of lipids beyond phosphatidylcholine (PC), including phosphatidylserine (PS) and sphingomyelin (SM). We note in the text (line 220) that 'phospholipid scramblases are generally unspecific' (this is indeed true for VDAC1 dimers and opsin, the two scramblases that we use in our study) and so our interest in the various NBD lipids is to identify those that may prove to be better reporters on account of a higher γ value, rather than to study specificity.

2.Sensitivity to other Scramblases
Can this system capture lipid scrambling by proteins with faster transport rates than VDAC1, such as members of the TMEM16 family? If there is a technical upper limit in rate detection, could it be extended—for example, by modulating vesicle size or lipid composition? Establishing the system's dynamic range would be crucial for defining its scope of applicability. Additionally, in line 432, the authors compare the VDAC1 kinetics obtained here with prior reports on TMEM16. Direct measurements of TMEM16 using the same assay platform would allow for a more meaningful comparison and strengthen the discussion (e.g., line 435). TMEM16 is also a favorable test case given its calcium-regulated activity, which could be used to test dynamic changes in scrambling under controlled conditions.

We agree that the TMEM16 scramblases are an attractive subject for our single vesicle assay. However, to our knowledge there is only one report of a functional fluorescently tagged TMEM16 protein – in Andra et al. (2018) J Biol Chem 293: 12248, we reported that ACP-tagged fungal nhTMEM16 is functional both as a channel and a scramblase. Unfortunately, the ACP reagents needed for tagging are not currently available and would require synthesis. The previous report of the activity of TMEM16F (Watanabe et al. (2018) PNAS 115: 3066) in a patterned supported bilayer, used bivalent antibodies to label the protein. This is not as feasible in our system and the extent to which the antibody interferes with function is not known.

Consequently, we turned to the GPCR opsin, the first scramblase to be functionally demonstrated via reconstitution of purified protein (Menon et al. (2011) Curr. Biol. 21:141). Ensemble measurements show that opsin has constitutive scramblase activity comparable to that of Ca^{2+}-activated nhTMEM16 and so would provide a good example of a fast scramblase for testing in our system. We labeled opsin with Alexa

Fluor 647 with 1:1 stoichiometry, reconstituted the labeled protein into vesicles and demonstrated its activity in ensemble measurements. Single vesicle assays on the reconstituted opsin sample are described in new Extended Data Figure 8, and associated Methods (line 660ff) and Results (line 453ff). The assays provided direct evidence that an opsin monomer is a scramblase, a result that could only be inferred previously from reconstitution statistics. Our analyses also indicated the power and limits of our assay. Individual vesicles with a single opsin protein showed a range of scrambling rates, with approximately a third of the sampled vesicles reaching the limit of measurement, i.e., the scrambling rate constant α was limited by the upper bound of the extraction rate constant γ, and so for these opsin monomers we could only set a lower limit of their unitary rate (>10,000 lipids per opsin per second). In the Discussion section (line 525ff) we offer suggestions whereby the upper bound for γ could be increased to enable improved kinetic resolution. However, we believe that converting these suggestions into practice is well beyond the scope of the current project.

3.Temperature Sensitivity
To what extent is the assay sensitive to temperature variations? While the scrambling rate itself is expected to be temperature-dependent, it would be helpful to know whether background activity—such as that from empty liposomes or non-crosslinked VDAC1—also varies with temperature. Understanding this would improve interpretability of the data and help establish optimal experimental parameters for future applications.

The temperature dependence of spontaneous flip-flop has been previously described and over a physiologically reasonable temperature range it is too slow to impact our measurements. Thus, we would not expect to see any changes in the readout from empty liposomes and proteoliposomes with NCL-VDAC1. The principal relevant temperature-dependent parameter in our assay is the desorption rate of NBD lipids. We now present data where we measure the rate in the range 24°C–40°C (new Extended Data Fig. 2g) and show that it increases with temperature as predicted. Indeed, the combination of parameter tests presented in Extended Data Figure 2f-h (flow rate, temperature, choice of reporter lipid) will help to further optimize experimental parameters for future applications as envisaged by the reviewer.

Reviewer #3 (Remarks to the Author):

Veit et al. report a single-vesicle assay for quantitatively measuring the kinetics protein-catalyzed lipid scrambling by single scramblase complexes. They reconstitute VDAC1-dimers in proteoliposomes and use a precise chemical crosslinking strategy to mimic the high VDAC1 concentration in the outer mitochondrial membrane, which would otherwise be difficult to recapitulate. These proteoliposomes are anchored on a solid support, and spiked with NBD lipids, either ion a symmetric or asymmetric distribution. Through a rather sophisticated combination of TIRF microscopy, lipid extraction through albumin excess and photobleaching, the authors are able to obtain kinetic traces from individual vesicles that contain only a single active scramblase complex.

These datasets – which I have not seen in such high quality – allow them to estimate the scrambling rates and amount of lipids scrambled per protein over time through kinetic modelling.

This high-throughput microscopy assay represents a significant step forward and is also a very timely contribution. There is quite a bit of evidence by now that lipid transbilayer movement is closely linked to inter-organelle lipid transport and thus the fundamental question of organelle membrane identity. Putting hard numbers on the rates of lipid transbilayer movement has never been important than today and the present manuscript goes a long way towards this goal. Therefore, I am in favor of publishing this

manuscript in NSMB.

Regarding specifics with regard to this manuscript, I have no issues with the biochemistry, the experimental design, which I like quite a lot, very cool biochemistry/quantitative microscopy. I do have some concerns, however, with regard to the kinetic modelling, where I am worried about a potential overfitting effect. This can be tested in a reasonably straightforward manner with little additional experimental effort.

We are naturally very pleased with the positive comments and specific appreciations of the reviewer!

Model fitting comment:

The kinetic model makes sense, and it may very well be that it is wholly appropriate to fit the data as the authors have done. I would, however would like to see a thorough testing of model choice and number of free parameters as I worry about the following:
In Figure 3c (single traces) and f (category averages) it appears that the single vesicle kinetic traces sort pretty much according to protein content which varies by a little more than one order of magnitude (if I see it correctly between 0 and 15 dimers per vesicle). The derived scrambling parameter alpha in contrast varies by three orders of magnitude. Taken these two things together, I would intuitively expect the following:

1. A very loose ordering of kinetic traces by protein concentration – much less pronounced to what was observed.

2. Higher standard deviation for kinetic traces derived from low-protein content vesicles compared to high protein-content vesicles, in particular for early timepoints. The former population captures the full activity distribution of very active and almost inactive dimers, whereas the latter have a much higher probability of containing at least one highly active dimer, which should then dominate the scrambling process. As far as I can see this trend is not observed.

There may very well be a reason that I am not aware off that may explain these observations, but the worry is always that the parameters become difficult to identify when there is not sufficient information in the kinetic traces (as is often the case with monotonously declining, noisy traces). It is possible that three free (but constrained) parameters allow for multiple combinations of parameter values that give a reasonable fit which could widen the observed distribution of values for alpha, in particular considering that these are single vesicle measurements, which are inherently noisy.

I would suggest the following:

1. I suggest deriving gamma from the asymmetric tail-labelled NBD PC population (if necessary, using a new dataset with higher time resolution) and using this as a fixed value in a model re-run for the traces contributing to h-j. These two models can then be compared using Akaike's information criterion or related approaches.
We used mock reconstituted vesicles instead (Fig. 3a) to obtain γ, as they most closely resemble the pCL-VDAC and opsin proteoliposome samples. We did not use the asymmetric tail-labeled NBD-PC vesicles as it is not possible to differentiate between vesicles that detach from the cover slip and those where the NBD-PC desorbs and is removed by buffer flow (using these vesicles would bias γ to higher values). We evaluated three different models, as explained in the Methods (line 739ff), Results (line 412ff) and

Extended Data Table 1, using Akaike and Bayesian Information Criteria (AIC, BIC), and chose Model 1 (with constraints on the range of α and γ) as it had the lowest AIC and BIC values.

2. Quite a few “single dimer traces” do not appear to fall outside of the NBD F/F0 range between 0.4 and 0.6 which is observed for empty vesicles, where scrambling should not take place. (i) In these cases, I would argue that it is unclear whether any scrambling takes place in the respective vesicle subpopulation of "single dimer" vesicles. (ii) How would model fits of this subset of traces compare to fits of the empty traces? To which extent would the distribution of alpha change if these traces were excluded? (iii) Related: It is possible that the initial constraint of the Lout/(Lin+Lout) ratio of 45-55% forces the model into scrambling when there is probably none, as this is less wide as the range actually observed for “empty” vesicles.
(i) We do not use the model for empty vesicles or vesicles with inactive scramblases as the model assumes that F/F_0 approaches 0 at $t=\infty$.
(ii) This is not an issue as we only fit the active traces.
(iii) Thank you for raising this point, as 0.4-0.6 is indeed more representative of the range for the $L_{out}/(L_{in}+L_{out})$ ratio we observe for empty liposomes. We have now re-analyzed the data using this range for the models tested in Extended Data Table 1.

3. Another possibility would be a bootstrapping approach – the authors could use subsets of kinetic traces to generate less noisy traces and gauge the variability in the kinetic parameters in this way.
We are not certain which traces the reviewer is suggesting should be combined, and any selection would be somewhat arbitrary. We think that because of the evaluation of fitting models presented in Extended Data Table 1, this approach would anyway now not be necessary.

I do believe that these re-analyses would make the paper much stronger – and since this is the best dataset on lipid scrambling activity that I am aware of, it will likely constitute a reference point for the literature going forward. Against that background I think that it is advisable to have a well-validated model choice in the paper.
We agree and thank you for your suggestions which prompted us to re-assess our analyses and improve the fitting model.

Minor comments

Figure 1d: The shape of theoretical traces does not look exponential – I’d expect an exponential decay of some sort, not a mostly linear function
Thank you – we have now replaced these theoretical traces with exponential decays.

I recommend to not use SDT as an abbreviation for sodium dithionite. The authors can use the chemical formula if they think an abbreviation is necessary (which may be needed in the figure), but I do not think that establishing yet another custom abbreviation helps the readability.
We now use dithionite or $[S_2O_4]^{2-}$ throughout; the term SDT has been removed.

Figure legend 2d: “This was followed by sodium dithionite addition to selectively bleach inner-leaflet NBD lipids”. I understand what the authors are trying to say here, but as a standalone sentence it is misleading.
This specific sentence has been rewritten as follows, and the entire description of panel **d** has been clarified: 'This was followed by dithionite ($[S_2O_4]^{2-}$) addition to bleach NBD-PC in the inner leaflet of VDAC1-containing vesicles (dithionite enters the vesicles via the VDAC1 channel).'

Figure 2e, Figure 3: I recommend not inverting the typical colormap of the fire lookup table – yellow usually encodes the highest values while violet encodes low values

Thank you for this suggestion. We played with this quite a bit and then settled on the one that we had originally presented, following the 'logic' that the more red/dark colors are easier to read as more protein. As we show the corresponding color bar next to it, interpretation should not be an issue.

NSMB-TR50979A

Text revisions in response to reviewers' comments.

Reviewer #1 (Remarks to the Author):
The authors have very well commented to all of my concerns and excellently revised the manuscript (especially through the additional experiments, data and figures). I suggest publication of this nice work.
RESPONSE: No revisions to the text are necessary.

Reviewer #2 (Remarks to the Author):
The authors have addressed most of the points we previously raised.

With respect to the reconstitution experiments, the authors chose opsin rather than TMEM16. However, the lipid-scrambling activity of VDAC1 and opsin has so far been evaluated primarily in reconstitution systems and has not been clearly demonstrated at the cellular level unlike TMEM16.

Although opsin is thought to be constitutively active, its lipid-scrambling activity has not, to our knowledge, been convincingly demonstrated in cells. Therefore, some uncertainty remains regarding the rationale for using opsin instead of TMEM16 (which is Ca^{2+}-stimulated but not constitutively active) as a reference scramblase.

Nevertheless, given that this work presents a platform technology for observing lipid scrambling at the single-vesicle level in cells, this reviewer believes that the revision has been carried out successfully.

RESPONSE: We note that in the original review comments, the reviewer suggested that we test a Ca2+-activated TMEM16 scramblase. We responded by agreeing that this was a good suggestion because of the opportunity to measure scrambling rates +/- Ca2+, but noted that no functional, fluorescently-tagged TMEM16 protein had been described, except in our previous work where we used an ACP tagging platform which is no longer available. Hence, we used opsin which we could label stoichiometrically with a fluorophore while retaining scrambling activity. The use of opsin serves the reviewer's original intent to demonstrate the limit of the assay in terms of discerning faster rates. TMEM16 proteins will undoubtedly become available for testing on our platform once their functional fluorescence tagging is achieved.

We are sensitive to the new point raised now by the reviewer that scrambling by opsin and VDAC-mediated has not been explicitly demonstrated at the cellular level – however, this is not relevant to our use of these proteins to demonstrate the capability of our technology to measure protein-mediated scrambling, a point that the reviewer appreciates in his/her final comments.

Reviewer #3 (Remarks to the Author):
The authors sufficiently addressed my comments in their revisions and I now recommend acceptance w/o any further revisions. Congratulations to a study well done!
P.S. I still think that inverting a very common color scheme (Fire Lut) is a debatable data visualization choice, but since this is a matter of taste/design, I shall not press the point.

RESPONSE: No revisions to the text are necessary.
